# Promotion of Bone Regeneration Using Bioinspired PLGA/MH/ECM Scaffold Combined with Bioactive PDRN

**DOI:** 10.3390/ma14154149

**Published:** 2021-07-26

**Authors:** Da-Seul Kim, Jun-Kyu Lee, Ji-Won Jung, Seung-Woon Baek, Jun Hyuk Kim, Yun Heo, Tae-Hyung Kim, Dong Keun Han

**Affiliations:** 1 Department of Biomedical Science, CHA University, 335 Pangyo-ro, Bundang-gu, Seongnam-si 13488, Gyeonggi-do, Korea; dptmf4011@cau.ac.kr (D.-S.K.); jklee2020@chauniv.ac.kr (J.-K.L.); jeongjiwon97@gmail.com (J.-W.J.); baiksw830@g.skku.edu (S.-W.B.); 1016jeffrey@gmail.com (J.H.K.); yun.heo@icloud.com (Y.H.); 2 School of Integrative Engineering, Chung-Ang University, 84 Heukseok-ro, Dongjak-gu, Seoul 06974, Korea; thkim0512@cau.ac.kr; 3 Department of Biomedical Engineering, SKKU Institute for Convergence, Sungkyunkwan University (SKKU), 2066 Seobu-ro, Jangan-gu, Suwon-si 16419, Gyeonggi-do, Korea; 4 Department of Intelligent Precision Healthcare Convergence, SKKU Institute for Convergence, Sungkyunkwan University (SKKU), 2066 Seobu-ro, Jangan-gu, Suwon-si 16419, Gyeonggi-do, Korea

**Keywords:** bone regeneration, poly(lactide-co-glycolide), magnesium hydroxide, extracellular matrix, polydeoxyribonucleotide, porous scaffold

## Abstract

Current approaches of biomaterials for the repair of critical-sized bone defects still require immense effort to overcome numerous obstacles. The biodegradable polymer-based scaffolds have been required to expand further function for bone tissue engineering. Poly(lactic-co-glycolic) acid (PLGA) is one of the most common biopolymers owing to its biodegradability for tissue regenerations. However, there are major clinical challenges that the byproducts of the PLGA cause an acidic environment of implanting site. The critical processes in bone repair are osteogenesis, angiogenesis, and inhibition of excessive osteoclastogenesis. In this study, the porous PLGA (P) scaffold was combined with magnesium hydroxide (MH, M) and bone-extracellular matrix (bECM, E) to improve anti-inflammatory ability and osteoconductivity. Additionally, the bioactive polydeoxyribonucleotide (PDRN, P) was additionally incorporated in the existing PME scaffold. The prepared PMEP scaffold has pro-osteogenic and pro-angiogenic effects and inhibition of osteoclast due to the PDRN, which interacts with the adenosine A_2A_ receptor agonist that up-regulates expression of vascular endothelial growth factor (VEGF) and down-regulates inflammatory cytokines. The PMEP scaffold has superior biological properties for human bone-marrow mesenchymal stem cells (hBMSCs) adhesion, proliferation, and osteogenic differentiation in vitro. Moreover, the gene expressions related to osteogenesis and angiogenesis of hBMSCs increased and the inflammatory factors decreased on the PMEP scaffold. In conclusion, it provides a promising strategy and clinical potential candidate for bone tissue regeneration and repairing bone defects.

## 1. Introduction

Bone fracture is the most common injury, which has high healing efficiency by oneself, but critical-sized bone fraction indispensably requires orthopedic surgery. To enhance the bone repair rate, various methods has been used, including autograft, allograft, xenograft, and artificial bone graft materials (e.g., tricalcium phosphate, hydroxyapatite, and bioglass). Among these treatments, autograft, obtained from the patient’s other position, is regarded as a ‘gold standard’ because of its high regeneration rate and superior osteoconductivity and osteoinductivity without any immune response [1,2,3]. Although autograft has numerous advantages including no risk of disease transfer, there are some limitations, such as restricted bone supply, donor site morbidity, and poor capability to accommodate defects. To conquer these hurdles, scaffold implantation is considered as an ideal way for bone tissue regeneration. Current bone tissue engineering (BTE) approaches still have numerous limitations such as low biocompatibility, mechanical property, osteoinductivity, and osteoconductivity. The polymer-based scaffold has been studied because of its biodegradability and biocompatibility. Among them, Poly(lactic-co-glycolic) acid (PLGA) was approved by the Food and Drug Administration (FDA) for diverse types of bone implants. However, it has been reported that its degradation byproducts, lactic acid and glycolic acid cause an acidic microenvironment at implanting site [4,5]. In previous studies, magnesium hydroxide (MH) performed outstanding pH neutralization ability for diverse tissue regeneration [6,7,8,9,10,11], in particular, bone repair [12,13,14]. However, MH as a hydrophilic inorganic molecule is difficult to disperse evenly in the hydrophobic polymer-based scaffold. To disperse metal ion molecule, in the prior study, the MH modified with a ricinoleic acid (mMH) was attempted to PLGA porous scaffold [9]. Plus, the incorporation of mMH into PLGA implant would be used to attenuate acid-induced inflammation triggered by the degradation products from the polymer and to improve the hydrophilicity of the scaffold.

As noted above, to overcome the limitation of the BTE scaffold, the extracellular matrix (ECM) isolated from mammalian tissues has been attempted in the scaffold for enhancing biocompatibility [15,16,17] and mimicking the natural composition of bone tissue. Especially, bovine-derived decellularized bone extracellular matrix (bECM), comprising mostly of calcium and phosphate, can improve not only biocompatibility also osteoconductivity of the scaffold.

Aside from these improvements, because bone repairing takes a longer time than other tissue in general, the ideal BTE scaffold should have an osteoinductive property. In this respect, in order to enhance osteoinductivity and bioactive function of the scaffold, polydeoxyribonucleotide (PDRN) was applied in bone regeneration. PDRN is a natural bioactive molecule normally extracted from salmon trout (*Oncorhynchus mykiss*) gonads, which is a short DNA form (50 to 2000 base pairs). Recently, some studies reported that PDRN has great effects on improving tissue regeneration since it plays as an adenosine A_2A_ receptor agonist. Adenosine A_2A_ receptor is a member of the G protein-coupled receptor (GPCR) family that has been proven as effective in improving angiogenesis and reducing inflammation [18,19,20]. Additionally, PDRN provides building blocks, nucleotides, and nucleosides to produce nucleic acids using less energy via the salvage pathway [21].

In this study, we designed a bioinspired scaffold by integrating mMH (M), bECM (E), and PDRN (P) into a porous PLGA (P) scaffold. We hypothesized that mMH could suppress the detrimental effect caused by PLGA degradation, reduce osteoclastogenesis; bECM could mimic the natural bone tissue microenvironment, improve osteoconductivity; PDRN could promote angiogenesis during bone repair. Therefore, the functionalized biodegradable PMEP scaffold would be applicable for effective bone regeneration with synergistic effects from these bioactive molecules.

## 2. Materials and Methods

### 2.1. Materials

Poly(D,L-lactide-co-glycolide) (PLGA, lactide:glycolide = 75:25, I.V. = 0.8–1.2) was purchased from Evonik Ind. (Essen, Germany). Magnesium hydroxide (MH), L-ascorbic acid, dexamethasone, and β-glycerophosphate were purchased by Sigma Aldrich (St. Louis, MO, USA). Ricinoleic acid was purchased from TCI product (Tokyo, Japan). The bovine bone-derived extracellular matrix powder (bECM; InduCera) was supplied by Oscotec Inc. (Seongnam, Korea). Polydeoxyribonucleotide (PDRN) was obtained from Goldbio (St. Louis, MO, USA). D-Plus™ cell counting kit 8 (CCK-8) cell viability assay kit was obtained from Dongin LS (Seoul, Korea).

### 2.2. Scaffold Preperation

The modified Mg(OH)_2_ was synthesized with ricinoleic acid (mMH) following the process with the previous study [9]. All scaffolds (PLGA, PME, and PMEP) were prepared by the freeze-drying method. In brief, the ice particles (200–300 μm) were prepared by spraying deionized water into liquid nitrogen as a porogen for the porous scaffold. A 20 wt% mMH and PDRN, and 50 wt% bECM (compared to PLGA) were mixed with 0.5 g of PLGA in 0.3 M dichloromethane solution. The mixtures and ice particles were stuffed into round PTFE mold (ø5 × 2 mm^2^). The filled molds were freeze-dried for 2 days to remove the ice and remaining organic solvent, then the porous scaffolds were obtained.

### 2.3. Scaffold Characterization

The cross-section morphology of the scaffolds was observed using scanning electron microscopy (SEM; GENESIS-1000, Emcraft, Gwangju, Korea). The thermal property of the scaffolds was analyzed by a thermal gravimetric analyzer (TGA 4000, PerkinElmer, Waltham, MA, USA). To assess the neutralization capacity of the scaffolds, the mass and pH changes were measured in 500 μL phosphate-buffered saline (PBS) solution (pH 7.4) with 20 µg/mL protease K (Bioneer, Daejeon, Korea) for 14 days. The inorganic compositions of the scaffold were measured using inductively coupled plasma-optical emission spectroscopy (ICP-OES, Optima 8000, PerkinElmer, Waltham, MA, USA). The water contact angle (WCA) was analyzed using the sessile drop method at room temperature to evaluate the hydrophilicity and hydrophobicity of the scaffolds.

### 2.4. Cell and Cytotoxicity Assay

Human bone-marrow mesenchymal stem cells (hBMSCs) were cultured in DMEM/low glucose media supplemented with 10% FBS (Hyclone, Logan, UT, USA) and 1% antibiotic–antimycotic solution (Gibco, Thermo Scientific Inc., Waltham, MS, USA). The cells were maintained under a humidified atmosphere with 5% CO_2_ at 37 °C. The viability and proliferation of the cells were determined using a Live-dead viability/cytotoxicity kit (Invitrogen, Thermo Scientific Inc., Waltham, MS, USA) and the fluorescence images were obtained using LSM880 (Zeiss, Jena, Germany) at 1, 3, and 7 days. The CCK-8 assay was conducted on the 3D scaffold at the same days.

### 2.5. Wound Healing Assay and Tubule Formation

The scratch wound healing assay was conducted to assess the migratory capacity of hBMSCs by PDRN. The cells were seeded into a 6-well culture plate at the density of 3 × 10^5^ cells/well and cultured for 1 day. The confluent wells were scratched, then washed with PBS solution. Cells were cultured with DMEM/low glucose containing 1% (*v*/*v*) FBS and added 100 μg/mL of PDRN. After 12 and 24 h, the plates were photographed and quantified the healed area using Image J software. To assess the angiogenic effects of the PDRN, 250 μL of matrigel matrix (Corning, Brooklyn, NY, USA) was added to pre-cooled 24-well plate and then incubated at 37 °C for 1 h. The human umbilical vein endothelial cells (HUVECs) were seeded onto coated well at the density of 1.2 × 10^5^ cells/well with EBM-2 (Lonza, Basel, Switzerland) containing 1% FBS, then added 100 μg/mL of PDRN. After 18 h, the cells were stained with calcein AM (C1430, Thermo Scientific Inc., Waltham, MS, USA), then photographed with a fluorescence microscope (U-RFL-T, Olympus, Tokyo, Japan). The tube length and branch point were quantified using Image J software.

### 2.6. RNA Extraction and Quantitative Real-Time PCR (qRT-PCR)

The RNA from scaffolds was extracted using Trizol reagent (15596018, Ambion, Invitrogen, Thermo Scientific Inc., Waltham, MS, USA) following the manufacturer’s instructions. The RNA concentration and quality were measured by spectrophotometer (ND-1000; Thermo Scientific, Waltham, MA, USA). The cDNA was synthesized using PrimeScript RT Reagent Kit (Perfect Real Time, Takara, Tokyo, Japan). The qRT-PCR was performed using each primer and SYBR Green PCR Master Mix (Applied Biosystems, Thermo Scientific Inc., Waltham, MS, USA). The expression of osteogenic, angiogenic, and inflammation-related genes was calculated with the 18S rRNA as a reference using the 2−ΔΔCt method. The primers used were as follows: 18S rRNA: forward, 5′-gcaattattccccatgaacg-3′ and reverse, 5′-gggacttaatcaacgcaagc-3′; IL6: forward, 5′-gatgagtacaaaagtcctgatcca-3′ and reverse, 5′-ctgcagccactggttctgt-3′; IL-1β: forward, 5′-tacctgtcctgcgtgttgaa-3′ and reverse, 5′-tctttgggtaatttttgggatct-3′; VEGF: forward, 5′-actggaccctggctttactg-3′ and reverse, 5′-tctgctccccttctgtcgt-3′; MMP2: forward, 5′-caccaccgaggattatgacc-3′ and reverse, 5′- cacccacagtggacatagca-3′; ALP: forward, 5′-atgaaggaaaagccaagcag-3′ and reverse, 5′-ccaccaaatgtgaagacgtg-3′; RUNX2: forward, ggtcagatgcaggcggccc-3′ and reverse, 5′-tacgtgtggtagcgcgtggc-3′; OCN: forward, 5′-cagcgaggtagtgaagagacc-3′ and reverse, 5′-tctggagtttatttgggagcag-3′.

### 2.7. Osteogenic Differentiation In Vitro

To assess the capacity of osteogenic differentiation on the 3D scaffold, hBMSCs were seeded onto the scaffold at the density of 5 × 10^5^ cells/scaffold. After 1 day, the medium was replaced with an osteogenic differentiation medium, DMEM/low glucose, containing 50 μM L-ascorbic acid, 0.1 μM Dexamethasone, and 10 mM β-glycerophosphate. After 7 days of osteogenic differentiation, the scaffolds were fixed with 10% formalin for 20 min, rinsed with deionized water, and stained with an alkaline phosphatase (ALP) staining kit (MK300, Takara, Japan). The stained samples were incubated in 15 and 30% sucrose solution in order, and embedded with frozen section media (FSC 22, Leica Biosystems, Wetzlar, Germany). The frozen samples were sectioned with a cryostat microtome (CM3050S, Leica Biosystems, Wetzlar, Germany). For the quantification of ALP activity, the scaffolds were lysed using an ALP assay kit (MK301, Takara, Tokyo, Japan). The assay was conducted according to the produced protocol.

### 2.8. Tartrate-Resistant Acid Phosphatase Staining and Activity

The osteoclastogenesis was identified by Tartrate-resistant acid phosphatase (TRAP) staining (MK-300, Takara, Tokyo, Japan). The RAW264.7 cells, mouse macrophage cell line, were seeded into a 24-well culture plate at the density of 2 × 10^4^ cells/well. After 1 day, 100 ng/mL of receptor activator of the NF-κB ligand (RANKL) was treated with RAW264.7 cells to induce differentiation into osteoclast. After 3 days, the scaffolds were put into trans-well inserts for co-culture with osteoclast. The TRAP staining was executed after 3 days.

### 2.9. Statistical Analysis

All experimental results were obtained through more than three independent experiments, and the values were described as mean ± standard deviation (SD). The statistical significance was analyzed by one-way ANOVA using Tukey’s post hoc method in GraphPad Prism 7.0 software [12] (GraphPad Software, Inc., San Diego, CA, USA). The statistically significant difference was defined as the *p* value being less than 0.05. The differences were considered significant when * *p* < 0.05, ** *p* < 0.01, *** *p* < 0.001, and # *p* < 0.0001.

## 3. Results and Discussion

### 3.1. Scaffold Characterization

**Figure 1:**
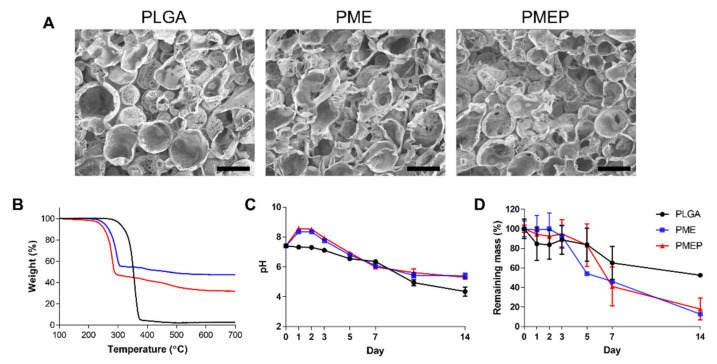
Scaffold characterization. (**A**) representative scanning electron microscopy (SEM) images of PLGA, PLGA/MH/ECM (PME), and PLGA/MH/ECM/PDRN (PMEP) scaffold. (Scale bars = 200 µm). (**B**) thermal gravimetric analysis (TGA) thermograms of each scaffold. Change of (**C**) pH and (**D**) mass during in vitro degradation in PBS solution with protease K at 37 °C for 14 days.

The biodegradable porous scaffolds containing PLGA, mMH, dECM, and PDRN were fabricated using the etching method with ice particles. In Figure 1A, the SEM images represent cross-section morphology that the pores of the scaffold were well-distributed and interconnected, so that the cells could easily attach and migrate in the scaffold during bone regeneration. Moreover, the 200–300 μm of porogens were used, that it is known as appropriate size for osteogenic differentiation in many other studies [22,23]. This size of porogen could be beneficial to cell ingrowth into the pore structures. The proportion of inorganic molecules in the scaffolds was analyzed by TGA (Figure 1B) and induced coupled plasma-optical emission spectroscopy (ICP-OES, Table 1). The PMEP scaffold consists of 195.44 ppm of magnesium, 265.74 ppm of calcium, and 151.20 ppm of phosphorus, respectively. The PME scaffold consists of 201.46 ppm of magnesium, 270.44 ppm of calcium, and 136.43 ppm of phosphorus, respectively. Interestingly, the amount of phosphorous in the PMEP was slightly higher than the PME because of the phosphate backbone in PDRN. The porous scaffolds with dissimilar surface roughness can cause different wettability and thus affect the permeability. The WCA was conducted to evaluate the wettability of the scaffold. The angles on PLGA, PME, and PMEP scaffold were 104.59, 93.99, and 77.12°, respectively. As bioactive molecules were added, the contact angles decreased. In other words, the PMEP scaffold has more hydrophilic property than the PLGA and PME ones.

The degradation of porous PLGA, PME, and PMEP scaffolds was observed in the presence of 20 µg/mL protease K for 14 days at 37 °C. The accelerative condition was conducted using protease K due to relatively high molecular weight of PLGA. In Figure 1C, pH value of the PLGA in PBS solution drastically decreased to 4.3 after 14 days of degradation. However, pH value of the solution containing the PME and PMEP specimens initially reached 8.3 and 8.5 and dropped slowly for 14 days to reach 5.5 and 5.4, respectively due to neutralization ability of mMH. The PME and PMEP scaffolds showed fast degradation performance than the PLGA only scaffold since those were containing numerous soluble bioactive molecules (Figure 1D).

### 3.2. Biocompatibility of the Scaffold

**Figure 2:**
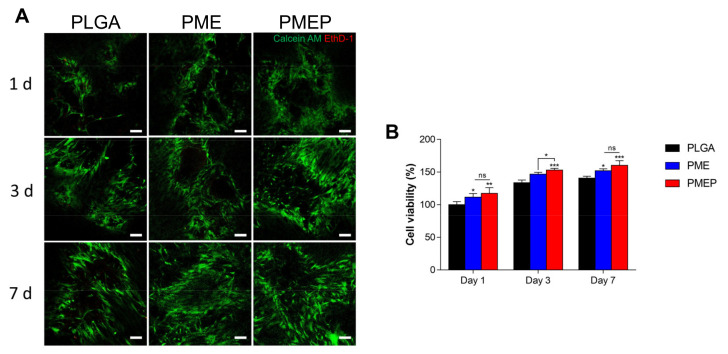
Biocompatibility of the scaffolds. (**A**) live-dead assay images on each scaffold at 1, 3, and 7 days (scale bar = 100 μm). (**B**) cell viability of the hBMSCs onto each scaffold at 1, 3, and 7 days in vitro. The differences were considered significant when ns = not significant (*p* ≥ 0.05), * *p* < 0.05, ** *p* < 0.01, and *** *p* < 0.001 (*n* ≥ 3).

To confirm cytotoxicity of the scaffolds in vitro, in Figure 2A, calcein AM and ethidium homodimer 1 (EthD-1) stainings were conducted with hBMSCs at 1, 3, and 7 days. Because of its well-known biocompatibility of PLGA, the EthD-1 positive cells indicating dead cells were observed rarely in all the scaffolds even the PLGA only group. What is more, the cells were observed evenly along with the pores of the scaffold. However, the population of calcein AM positive cells, the live cells, was getting increased in the PME and the PMEP than the PLGA at 1, 3, and 7 days, respectively. In Figure 2B, the cell viability was quantified using CCK-8 in 1, 3, and 7 days. The initial adhesion rate of hBMSCs on the PMEP significantly increased due to its surface hydrophilicity for cell recruitment (*p* < 0.01). Because a hydrophilic surface promotes the adhesion of the cells [24], the initial cell adhesion rate significantly increased on the PMEP scaffold.

As mentioned previously, the PLGA produced acidic byproducts during hydrolytic degradation, so that the cell slightly proliferated for 7 days. However, the cell viability on the PME and particularly, the PMEP scaffold was remarkably enhanced for 7 days, *p* < 0.05 and *p* < 0.001, respectively. Consequently, the incorporation of mMH, bECM, and PDRN could constrict the adverse effect on cell cytotoxicity caused by hydrolytic degradation of PLGA.

### 3.3. Confirmation of Angigenic Ability

Angiogenesis is a physiological process by which new blood vessels form from the pre-existing vascular network, allowing the delivery of oxygen and nutrients to the body’s tissues. Angiogenesis has been studied as a therapeutic target in regenerative medicine. Bone is also richly vascularized tissue, so that new blood vessels play a critical role in maintaining the bone cells survival and stimulating their activity. However, in situ vascularized bone regeneration still remains in the extreme challenge [25,26,27,28].

**Figure 3:**
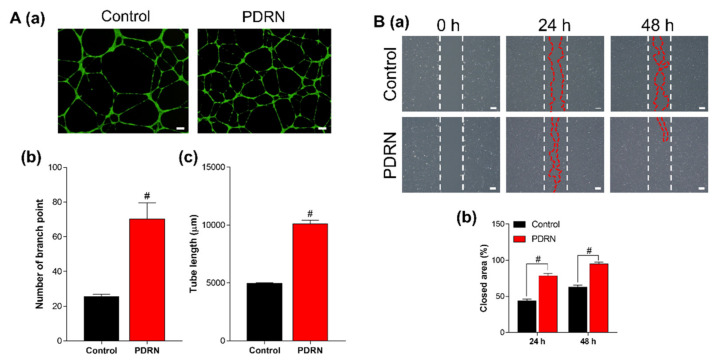
Biological effects of PDRN. (**A**) tubule-forming assay; images of HUVECs stained with calcein AM (scale bar = 200 μm) (**a**) and quantification of branch point (**b**) and tube length (**c**). (**B**) wound healing assay; optical images (scale bar = 200 μm) (**a**) and quantification of closed area at 24 and 48 h (**b**). The differences were considered significant when # *p* < 0.0001 (*n* ≥ 3).

As mentioned previously, PDRN has a pro-drug activity carried out through two different mechanisms. First, PDRN supplies purines and pyrimidines, promoting DNA synthesis or repair through the ‘salvage pathway’ [18,21]. Next, PDRN stimulates adenosine A_2A_ receptor, as suggested by Thellung et al. studied the effect of PDRN using 3,7-dimethyl-1-propargylxanthine (DMPX), a selective adenosine A_2A_ receptor antagonist [29]. Adenosine and adenosine A_2A_ receptor were considered clinically important to enhance angiogenesis. Wang et al. studied that adenosine enhances cell growth and induces tube formation in HUVECs in vitro [30]. In Figure 3, the biological ability of PDRN was investigated in angiogenesis and wound healing for effective bone tissue repair. When PDRN treated, HUVECs had formed a significant number of branch points and longer lengths of tubes. On the same side of Figure 2, because PDRN could enhance the growth and migratory ability of hBMSCs, the wound closure rates also highly increased to 34.8 and 31.9% in PDRN treated groups compared to control at 24 and 48 h, respectively. To conclude, these outstanding biological abilities of PDRN give a synergistic effect to achieving a novel strategy for bone regeneration.

### 3.4. Biological Abilities of the PMEP Scaffold with hBMSCs: Anti-Inflammation and Angiogenesis

**Figure 4:**
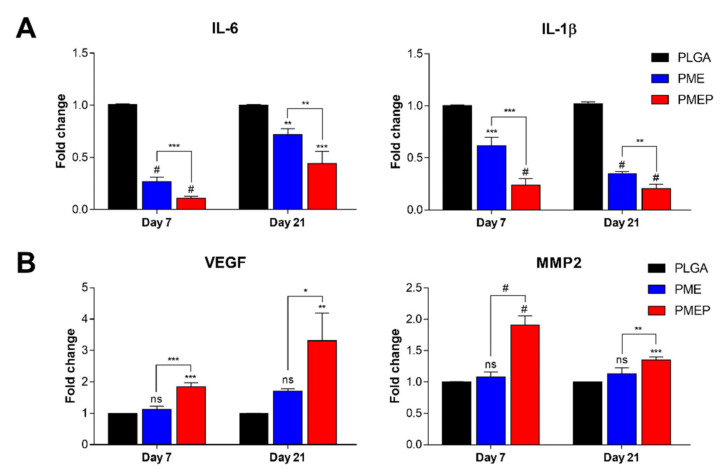
Anti-inflammatory and angiogenic effects on the scaffolds using hBMSCs. Gene expressions of hBMSCs onto the scaffolds related to (**A**) anti-inflammation: IL-6 and IL-1β, and (**B**) angiogenesis: VEGF and MMP2 at 7 and 21 days. The differences were considered significant when ns = not significant (*p* ≥ 0.05), * *p* < 0.05, ** *p* < 0.01, *** *p* < 0.001, and # *p* < 0.0001 (*n* ≥ 3).

Since mineralization is affected by numerous mechanisms, biomaterials should have a variety of functions, such as vascularization, inhibition of inflammation, as well as osteogenesis to reach effective bone regeneration. The quantitative real-time PCR (qRT-PCR) was conducted to determine the expression of inflammation and angiogenesis-related genes on 3D scaffolds with hBMSCs. The effect of the scaffolds was assessed in osteogenic media at 7 and 21 days. As shown in Figure 4A, the PME scaffold restricted the expression of inflammatory genes, interleukin-6 (IL-6) and interleukin-1β (IL-1β), compared to the PLGA scaffold. Plus, the PMEP scaffold effectively suppressed the above-mentioned gene expression even in comparison with the PME one. In recent studies, the researchers demonstrated that the PDRN affects to increase expression of vascular endothelial growth factor (VEGF) and to suppress the production of pro-inflammatory cytokines by stimulating the A_2A_ receptor [18,21,31]. As a result, the PDRN could promote angiogenesis and inhibit inflammation during bone repair. In Figure 4B, the PME scaffold exhibited a negligible difference in the expression of angiogenesis-related genes, including VEGF and matrix metalloproteinase-2 (MMP2). Likewise, the PMEP scaffold promoted the highest gene expression of VEGF and MMP2 on both days. It is notable that the addition of PDRN on the scaffold has effects on not only reducing the inflammatory response but also significantly enhancing vascularization. In prior analysis (Figure 3), we confirmed the effectiveness of PDRN, treated directly in cells in the 2D environment. Further, incorporation of mMH, bECM, and PDRN in the biodegradable porous 3D scaffold displayed attenuating inflammatory response and enhancing angiogenesis, simultaneously. These results suggest that the PDRN effect is not only for angiogenesis but also may influence several factors containing the healing process.

### 3.5. Induction of Osteogenesis in 3D Scaffold

**Figure 5:**
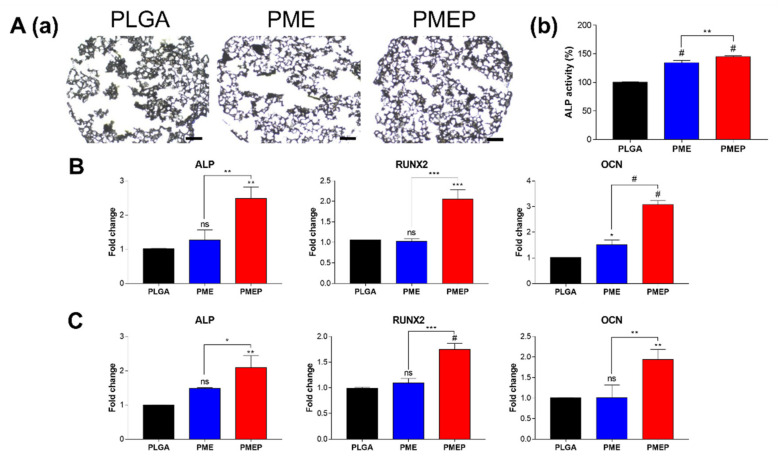
Osteogenic differentiation onto scaffolds using hBMSCs. (**A**) optical images of the scaffolds stained with ALP for 7 days in osteogenic medium (**a**). Scale bars indicate 400 μm. What is more, the quantification of ALP activity onto each scaffold (**b**). (**B**,**C**) gene expressions of hBMSCs related to osteogenesis onto the scaffolds; ALP, RUNX2, and OCN at 7 and 21 days of osteogenic differentiation. The differences were considered significant when ns = not significant (*p* ≥ 0.05), * *p* < 0.05, ** *p* < 0.01, *** *p* < 0.001, and # *p* < 0.0001 (*n* ≥ 3).

To identify the osteogenic capacity of the scaffolds, hBMSCs were seeded onto the scaffold. ALP is known as an early marker of osteogenesis. After 7 days of osteogenic differentiation, the ALP staining was conducted on each scaffold. Figure 5A showed that the PMEP scaffold formed more degrees of staining with less collapsing of internal structure compared to other scaffolds. Moreover, the PMEP scaffold enhanced ALP activity, which was even significantly higher than the PME one. These results implied that the PME scaffold could induce osteogenic differentiation of hBMSCs effectively, and by adding PDRN, the osteogenesis was more enhanced. Further investigation of cell differentiation was verified through gene expression analysis of the osteogenic markers. The expression of osteogenesis-related genes including ALP, runt-related transcription factor 2 (RUNX2), and osteocalcin (OCN) was also evaluated at 7 (Figure 5B) and 21 days (Figure 5C). In general, the RUNX2 and OCN are, respectively, used as mid- and late-responsive genes for bone formation. The results exhibited that the PMEP scaffold significantly up-regulated ALP, RUNX2, and OCN at all days. The mRNA expression of genes in the PMEP scaffold respectively increased by 2.48-, 2.05-, and 3.07-fold higher than the PLGA group at 7 days. The expressions on 21 days were also up-regulated by 2.08-, 1.75-, and 1.94-fold higher in the PMEP scaffold, respectively. These results indicated that the MH provides biocompatibility, bECM has osteoconductivity, and the PDRN promotes angiogenesis and osteogenesis by stimulating the A_2A_ receptor. In conclusion, the PMEP scaffold has the potentials that not only effectively induce early-stage of osteogenesis, but also affect the maturation of hBMSCs for bone regeneration.

### 3.6. Attenuation of Osteoclastogenesis

**Figure 6:**
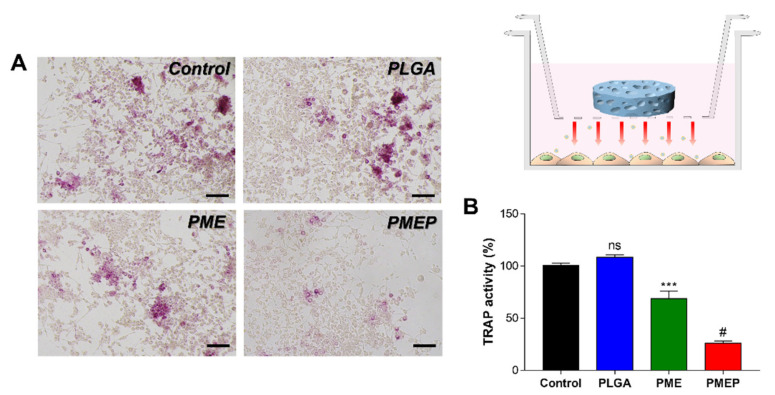
RANKL-induced osteoclastogenesis of RAW264.7 cells for 3 days. Experimental design of osteoclastogenesis using porous scaffold (right above). (**A**) optical images of TRAP+ cells (scale bar = 100 μm). (**B**) quantification of TRAP activity. The differences were considered significant when ns = not significant (*p* ≥ 0.05), *** *p* < 0.001, and # *p* < 0.0001 (*n* ≥ 3).

Recently, to develop the bone repairing materials, one of the most important issues is bone homeostasis between osteoclasts and osteoblasts, because excessive differentiation of osteoclasts affects bone tissue resorption, which would occur metabolic bone-related diseases such as osteoporosis [32,33,34]. In general, when bone fracture occurs, both osteoblasts and osteoclasts are activated. Immoderate osteoclastogenesis and osteoblastogenesis cause eventually the delay of bone formation or nonunion. Thus, the BTE scaffold should control initial immoderate osteoclastogenesis, which is critical for promoting osteoblast activity and enhancing bone mineral density [35,36,37]. To evaluate the control ability of the 3D scaffold, we designed an indirect co-culture system (Figure 6) using a trans-well insert. In Figure 6A, optical images represent TRAP positive cells after 3 days of RANKL treatment. The stained cells (purple) significantly decreased in the PMEP group compared to the control, the PLGA and the PME. To quantify the osteoclast activity, TRAP activity was analyzed with the 3D scaffolds. In the PLGA group, the activity slightly increased in comparison to RANKL treated control group. However, the secreted bioactive molecules from the PME and PMEP scaffold attenuated RANKL-induced differentiation into osteoclast of RAW264.7 cells for 31.7 and 74.4%, respectively, than control. Overall, the PMEP scaffold has multifunctional abilities in inhibition of local inflammation, promotion of angiogenesis, and attenuation of osteoclastogenesis. The bioinspired PMEP scaffold would be clinically utilized as a bone grafting material for tissue regeneration of various sizes and shapes.

## 4. Conclusions

Because of their biodegradability and biocompatibility in physiological environments, biodegradable synthetic polymers are commonly used for a wide range of biomedical applications, especially bone repairing. Among them, PLGA has been clinically used as a bone grafting material. However, PLGA-based bioimplants often occur clinical failure due to low mechanical property and local acidification. Our findings proposed that mMH could enhance the mechanical property and neutralize acidification. The bECM was introduced to improve osteoconductivity by providing natural calcium and phosphate rich environment. Additionally, the DNA-derived bioactive molecule, PDRN facilitated biocompatibility and in situ vascularization during osteogenesis.

Taken together, we investigated that the synergistic interaction of mMH, bECM, and PDRN in the PMEP scaffold. The bioactive PMEP scaffold could inhibit osteoclastogenesis and promote adequate cell proliferation, angiogenesis, and osteogenesis in vitro. In the future study, we expect that the PMEP scaffold can regenerate the new bone in vivo by multifunctional abilities. This versatile biodegradable scaffold would apply to a novel bone tissue engineering as an advanced biomedical device.

## Figures and Tables

**Table 1:** ICP-OES and water contact angle.

Group	Mg (ppm)	Ca (ppm)	P (ppm)	Water Contact Angle (°)
PLGA	-	-	-	104.59 ± 4.24
PLGA/mMH/bECM (PME)	201.46 ± 0.93	270.44 ± 2.53	136.43 ± 0.94	93.99 ± 9.90
PLGA/mMH/bECM/PDRN (PMEP)	195.44 ± 3.03	265.74 ± 5.75	151.20 ± 1.60	77.12 ± 4.49

## Data Availability

The data presented in this study are available on request from the corresponding author.
